# Advancements in Research on Mesenchymal Stem-Cell-Derived Exosomal miRNAs: A Pivotal Insight into Aging and Age-Related Diseases

**DOI:** 10.3390/biom14111354

**Published:** 2024-10-24

**Authors:** Minglei Huang, Ye Liu, Longze Zhang, Shuangmin Wang, Xianyao Wang, Zhixu He

**Affiliations:** 1 Department of Immunology, Zunyi Medical University, Zunyi 563000, China; 15180712615@163.com (M.H.); liuye19991023@163.com (Y.L.); 19982633612@163.com (S.W.); 2 Scientific Research Center, The First People’s Hospital of Zunyi (The Third Affiliated Hospital of Zunyi Medical University), Zunyi 563000, China; zlongze@163.com; 3 Collaborative Innovation Center of Tissue Damage Repair and Regeneration Medicine, Zunyi Medical University, Zunyi 563000, China

**Keywords:** mesenchymal stem cells, exosomes, miRNAs, aging, aging-related diseases

## Abstract

Mesenchymal stem cells (MSCs) are capable of differentiating into various cell types and play a crucial role in repairing aging tissues and diseased organs. Aging manifests as a gradual loss of cellular, tissue, and organ function, leading to the progression of pathologies. Exosomes (Exos) are extracellular vesicles secreted by cells, which maintain cellular homeostasis, clear cellular debris, and facilitate communication between cells and organs. This review provides a comprehensive summary of the mechanisms for the synthesis and sorting of MSC–Exo miRNAs and summarizes the current research status of MSCs–Exos in mitigating aging and age-related diseases. It delves into the underlying molecular mechanisms, which encompass antioxidative stress, anti-inflammatory response, and the promotion of angiogenesis. Additionally, this review also discusses potential challenges in and future strategies for advancing MSC–Exo miRNA-based therapies in the treatment of aging and age-related diseases.

## 1. Introduction

The aging of the global population has emerged as a significant public health concern, with projections indicating that by 2050, the proportion of individuals aged 65 and older will reach 38% [1]. Population aging has become a significant public health issue. Aging is recognized as a leading risk factor for many diseases, with approximately two-thirds of global daily deaths linked to age-related conditions [2]. One of the detrimental effects of aging is immunosenescence, characterized by a decline in the body’s adaptive capacity to external factors with advancing age, leading to an increased susceptibility to diseases [3]. Aging organisms accumulate senescent cells, which secrete a complex set of pro-inflammatory cytokines through autocrine and paracrine signaling, inducing a senescent phenotype in neighboring cells. This process leads to systemic chronic inflammation or these cells can carry pro-senescence molecules into other cells via exosomes, disrupting the cellular microenvironment and ultimately contributing to the development of aging-related diseases [4,5]. Therefore, studying the mechanisms related to organismal aging and identifying new anti-aging strategies have become urgent priorities.

In recent years, MSCs have garnered significant attention due to their immunomodulatory effects, migratory capacity, and regenerative potential. MSC-based cell therapies have demonstrated clinical feasibility and safety, but there is a lack of reliable methods to assess the immunosuppressive effects of MSCs [6]. Extensive research indicates that the therapeutic efficacy of MSC therapy does not depend on the implantation of MSCs at the injury site or their differentiation capacity, but rather on paracrine mechanisms involved in intercellular communication [7]. MSCs engage in close interactions with adjacent cells via direct intercellular contact, along with the secretion of paracrine signaling molecules and extracellular vesicles [6]. Studies have confirmed that exosomes secreted by MSCs possess anti-aging properties [8,9] and play a role in regulating the progression of aging-related diseases, such as cardiovascular diseases, diabetes, and cancer.

miRNAs are widely present in organisms and regulate multiple target genes, influencing signaling pathways and thereby affecting the occurrence and progression of diseases [10]. In recent years, various small-molecule drugs targeting the regulation of miRNAs, such as miRNA inhibitors and mimics, have been developed for disease treatment, with their delivery to diseased sites facilitated through nanocarriers [10,11]. The use of miRNAs as therapeutic agents for disease treatment has already entered the clinical trial phase [12]. In 2007, it was first discovered that exosomes could transport miRNA between cells, highlighting the crucial role of miRNA in the function and transport mechanisms of exosomes [13]. Therefore, exploring the biological role of MSC–Exo miRNAs in cellular communication is of great significance for deepening our understanding of aging and aging-related diseases. This paper primarily analyzes the relationship between MSC–Exo miRNAs and aging, as well as aging-related diseases, providing a supportive framework for the diagnosis and treatment of these conditions.

## 2. MSCs, Exos, and miRNAs

### 2.1. Biogenesis and Composition of Exosomes

MSCs are multipotent progenitor cells capable of differentiating into various lineages, including osteoblasts, chondrocytes, adipocytes, and others. They can be extracted from bone marrow, adipose tissue, the umbilical cord, placenta, and other tissues [14,15]. MSCs possess powerful immunomodulatory capabilities and regenerative properties, making them one of the most commonly used stem cells in cell therapy trials [16]. MSCs can exert immunomodulatory effects by either direct interaction with immune cells or through paracrine pathways, secreting a variety of cytokines, growth factors, and chemokines [17]. Regulating the metabolic activity of MSCs using stimuli such as inflammatory environments, hypoxia, and heat shock can subsequently influence the immunomodulatory capacity of MSCs [17,18]. Furthermore, the immunoregulatory capabilities of MSCs vary based on their source [19]. Research has identified several mechanisms by which MSCs treat diseases, including the following: (1) secreting proteins/peptides and hormones; (2) transferring mitochondria through various pathways; and (3) transferring exosomes or microvesicles that contain RNA, proteins, and other substances [20,21].

**Figure 1:**
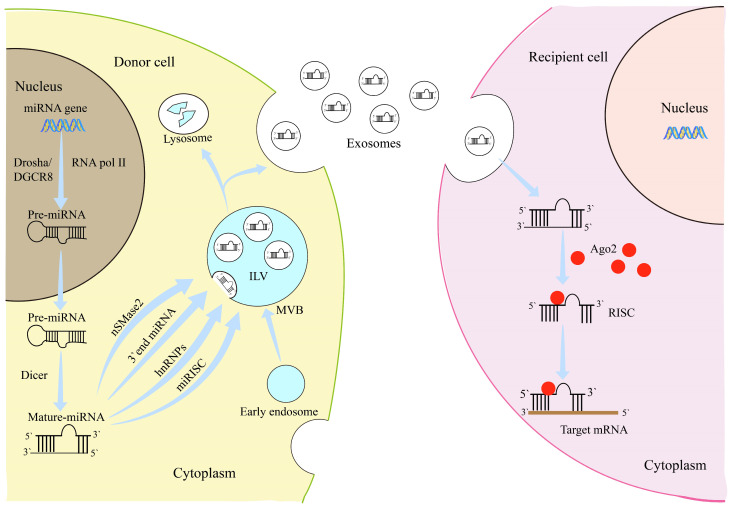
Generation of exosomes and sorting of microRNAs. The primary miRNA gene is transcribed by RNA polymerase II and processed by DGCR8 and Drosha enzymes to generate pre-miRNA. The pre-miRNA is then exported from the nucleus to the cytoplasm, where it is cleaved by Dicer and unwound by helicase to produce mature miRNAs. miRNAs are sorted into exosomes via four distinct pathways and delivered to target cells through exosomes. Upon reaching the target cells, miRNAs bind to target genes and regulate their expression levels. Figure 1 was drawn by the author of this article.

MSCs can secrete microvesicles and exosomes through paracrine pathways [22,23,24]. Exos are extracellular vesicles measuring roughly 30–150 nm in diameter and were first discovered in sheep reticulocytes. By transporting proteins, lipids, and nucleic acids, Exos play a crucial role in maintaining cellular homeostasis, clearing cellular debris, and facilitating communication between cells and organs [25,26]. As shown in Figure 1, the generation of exosomes involves four steps: budding, invagination, multivesicular body formation, and secretion. The cell membrane invaginates to form an endosome covered by clathrin, which then buds off to form an early endosome in the cytoplasm [27,28]. The “cargo” to be released accumulates in the central vesicular region of the early endosome and participates in the maturation process of the endosome. Subsequently, the endosomal membrane invaginates to form intraluminal vesicles (ILVs), which eventually evolve into multivesicular bodies (MVBs). MVBs have two possible fates: they can either fuse with lysosomes, leading to the degradation of the vesicular contents, or merge with the plasma membrane, releasing the contents into the extracellular space to form exosomes [27,29]. Exos from different sources of MSCs share many similarities in biological functions. They can carry various molecules and protect their cargo from degradation in the bloodstream, making them a promising therapeutic approach [25,30].

### 2.2. Sorting of miRNAs in Exosomes

MiRNAs are small non-coding RNAs that bind to the 3′ untranslated regions (3′ UTRs) of target genes to regulate their expression, thereby influencing various biological processes such as cell growth, differentiation, and metabolism [31,32]. They can also bind to the coding regions or 5′ UTRs of mRNA molecules to inhibit protein expression [33]. The classic pathway for miRNA synthesis is a complex protein process. Initially, primary miRNA (pri-miRNA) is transcribed by RNA polymerase II (RNA pol II) and contains one or more hairpin structures. This is then processed by the Drosha enzyme and its cofactor DGCR8 into a precursor miRNA (pre-miRNA) of 70–100 nt, which is transported to the cytoplasm. Finally, the Dicer enzyme and helicase further process the pre-miRNA into a mature miRNA. This mature miRNA binds with Argonaute (Ago2) protein to form the RNA-induced silencing complex (miRISC), which then binds to mRNA to regulate the expression of target genes [34,35]. miRNAs that migrate to the extracellular space and enter bodily fluids are referred to as circulating miRNAs. Research indicates that approximately 90% of circulating miRNAs are associated with protein complexes such as Ago2, nucleophosmin 1, and high-density lipoproteins, with the remaining 10% being secreted while encapsulated within Exos [35,36].

Before exosomes bud from the cellular membrane, they need to recruit the appropriate “cargo” into their structure. Since this “cargo” consists of various types of molecules, such as proteins, lipids, circular RNAs, and miRNAs, the sorting mechanisms for these components within exosomes are also different [37]. In recent years, miRNAs have been identified as key active components of exosomes. As shown in Figure 1, there are four main pathways through which miRNAs may enter exosomes. The first is the neutral sphingomyelinase 2 (nSMase2)-dependent pathway, where nSMase2 mediates the sorting of miRNAs, and α2,6-sialylation can enhance nSMase2 activity [38,39]. The second pathway is the 3′ end-dependent pathway, where the 3′ end of the miRNA sequence may carry a critical sorting signal that helps incorporate it into exosomes [40]. The third pathway is the heterogeneous nuclear ribonucleoprotein (hnRNP)-dependent pathway, where hnRNPA2B1 binds to specific motifs (GGAG/CCCU) to facilitate miRNA transport. Zhao et al. revealed that the hnRNPA2B1 protein mediates the packaging of miR-934 into exosomes secreted by cells, which are then transferred to macrophages [41]. In addition, mutating specific miRNA sequences can enhance the miRNA content in Exos [42]. The final pathway involves miRISC, and research has shown that exosomes co-localize with key components of miRISC [43,44].

## 3. MSCs–Exos and Aging

Aging is the gradual loss of function in tissues and organs over time. The hallmarks of aging can be summarized into the following three criteria: (1) hallmarks change in parallel with aging; (2) experimental evidence demonstrates that hallmarks can accelerate the aging process; and (3) therapeutic interventions targeting hallmarks can delay or reverse aging [45]. Aging can be caused by various factors, including telomere shortening and damage, DNA damage, oxidative stress, and the inactivation of tumor suppressor genes [46]. Cellular senescence can lead to changes in the senescence-associated secretory phenotype (SASP), which includes the secretion of pro-inflammatory factors, chemokines, matrix metalloproteinases (MMPs), and growth factors [47,48]. The SASP is a defining feature of senescent cells, driving their pathophysiological impact by promoting and propagating cellular senescence via autocrine and paracrine signaling pathways, as well as initiating immune responses that facilitate the clearance of senescent cells [49].

Oxidative-stress-induced aging is caused by the accumulation of reactive oxygen species (ROS) in the body, leading to tissue and organ damage and loss of function. Prolonged elevated ROS levels contribute to the development of age-related diseases [50]. ROS encompass various chemical species, including superoxide anions, hydroxyl radicals, and hydrogen peroxide. Intracellular ROS are primarily generated by mitochondria. During electron transport, NADH dehydrogenase and ubiquinone-cytochrome c reductase release superoxide anions (O^−^_2_), which subsequently form superoxide radicals [51]. Another pathway for ROS production involves the cytochrome P450-catalyzed process. Due to the low efficiency of NADPH in transferring electrons through electron carriers to P450 for oxidation reactions, ROS are generated during this process [52]. MSCs–Exos alleviate cellular senescence by participating in the reduction in oxidative stress. Chen et al. co-cultured cholangiocyte organoids with human placental MSCs–Exos and induced oxidative-stress-like senescence in the cells using H_2_O_2_. The results showed that the MSC–Exo group could delay the senescence of cholangiocyte organoids, reduce the expression of senescence markers P16, P21, and β-galactosidase, and suppress the secretion of the SASP [9]. After intravenous injection of MSCs–Exos into aging mice, a significant improvement in the morphology, structure, and arrangement of hippocampal neurons was observed compared to the PBS-treated group, with a marked reduction in the proportion of degenerated cells and a notable enhancement in memory function. Measurements of the ROS levels in the mice revealed a reduction in ROS levels in the MSC–Exo-treated group [8]. Zhang et al. further investigated the mechanism by which MSCs–Exos delay aging, revealing that MSCs–Exos inhibit the sirtuin 1 (SIRT1) and p53 signaling pathway to slow down the aging process [8].

Inflammation is a major consequence of DNA damage and a hallmark of aging, associated with various aging-related diseases [53]. Senescent cells promote inflammation and tumor progression in neighboring cells through the secretion of a range of pro-inflammatory factors [54]. Studies have shown that MSCs–Exos possess potent anti-inflammatory properties, capable of mitigating inflammation both in vitro and in vivo [55,56]. MSC–Exos exert protective effects on aging organisms by reducing markers of senescence and genomic instability. A comprehensive omics analysis by Ling et al. demonstrated that umbilical-cord-derived MSCs–Exos protect the livers of aging mice. A metabolomic analysis revealed that MSCs–Exos effectively reduced lipotoxicity and the production of inflammation-related lipid molecules, while a phosphoproteomic analysis showed a significant reduction in the phosphorylation levels of fatty-acid-associated proteins [57]. In the osteoarthritis microenvironment, MSCs–Exos exert antigen-specific anti-inflammatory effects by inhibiting T lymphocyte proliferation, reducing the proportion of CD4 and CD8 T cell subsets, and downregulating the expression levels of interleukin 6 (IL-6) and interleukin 1β (IL-1β) [58]. Liu et al. found that bone-marrow-derived MSCs–Exos can induce M1 and M2 polarization of microglial cells, inhibiting NLRP3-mediated inflammation [59]. MSCs–Exos suppress inflammatory responses by inhibiting the expression levels of pro-inflammatory factors, such as tumor necrosis factor-alpha (TNF-α), interleukin 6 (IL-6), and interleukin 8 (IL-8), while promoting the expression of anti-inflammatory factors, including CD163, interleukin 10 (IL-10), CD206, transforming growth factor beta 1 (TGFβ1), and arginase 1 (ARG1) [60].

Additionally, a clinical study found that injecting exosomes derived from adipose tissue stem cell secretions into one side of the face of participants, with saline injected into the other side, effectively improved skin wrinkles, elasticity, hydration, and pigmentation, with no adverse effects observed [61]. In summary, during the aging process, oxidative stress and inflammation interact and mutually promote each other, forming a complex network [62]. MSC-derived exosomes exert protective effects in the regulation of both oxidative stress and inflammation. These studies suggest that MSCs–Exos possess significant anti-aging potential. Investigating their anti-aging mechanisms is crucial for developing therapies targeting aging-related diseases.

## 4. MSC-Derived Exosomal miRNAs in Aging

**Figure 2:**
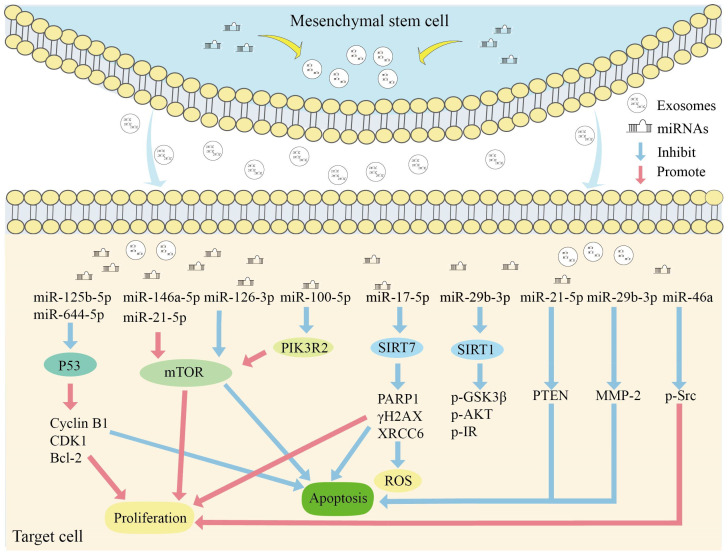
The role of MSC–Exo miRNAs in aging. MSCs–Exos regulate target genes and aging-related signaling pathways, such as the P53, mTOR, and sirtuins pathways, through targeted delivery of miRNAs. This modulation promotes cell proliferation, inhibits apoptosis and ROS levels, and exerts protective effects during the aging process and disease development. Figure 2 was drawn by the author of this article.

Studies have shown that the expression of miRNAs in MSCs–Exos undergoes significant changes and plays a role in regulating aging through various mechanisms [63]. Through miRNA expression profiling and the molecular functional characterization of MSCs–Exos, it was found that most miRNAs play significant roles in regulating cell death pathways, aging, T cell differentiation, stem cell regulation, and hematopoiesis [64]. Studies further indicate that MSCs–Exos can improve the aging conditions of cells, tissues, and organs both in vitro and in vivo [65,66,67]. MSCs–Exos regulate aging-related signaling pathways through miRNA modulation, playing a role in the regulation of aging and aging-associated diseases (Figure 2).

### 4.1. p53 Signaling Pathway

The p53 gene is involved in regulating various biological processes, such as the cell cycle, apoptosis, and DNA damage repair [68]. DNA damage repair is a crucial mechanism for preventing the survival and proliferation of cells undergoing malignant transformations. Its dysfunction leads to the accumulation of damage, tissue dysfunction, and degeneration, thereby accelerating the aging process [69]. In a mouse model of acute kidney injury, an intravenous injection of human umbilical cord-derived MSCs–Exos resulted in a significant accumulation of exosomes at the site of renal injury. Compared to the control group, the MSC–Exo treatment alleviated kidney injury in mice and promoted renal tubular epithelial cell proliferation. A further analysis revealed that MSCs–Exos carry miR-125b-5p, which interacts with p53, inhibiting p53 signaling and preventing G2/M phase arrest [70]. Cao et al. found that human umbilical-cord-derived MSCs–Exos can effectively inhibit the senescence-associated phenotype induced in chondrocytes by osteoarthritis, restoring cellular activity [71]. The p53 signaling pathway is key in this process, with MSCs-Exos delaying senescence and restoring the chondrocyte phenotype by inhibiting the activation of p53. Sequencing of MSCs–Exos identified 17 upregulated miRNAs whose target genes directly participate in pathways that promote senescence [71]. Hypoxia-induced MSCs–Exos can reduce the infarct size in myocardial infarction and improve cardiac function. miR-125b-5p is significantly enriched in hypoxia-induced MSCs–Exos, and its expression inhibits cardiomyocyte apoptosis by suppressing p53 expression, thereby protecting cardiomyocytes [72]. In an ovarian insufficiency model, treatment with bone-marrow-derived MSCs–Exos effectively improved follicular morphology in mice. Further research revealed that MSCs–Exos downregulated P53 expression via miR-664-5p, inhibiting the expression of apoptosis-related proteins [73].

### 4.2. mTOR Signaling Pathway

The mechanistic target of rapamycin kinase (mTOR) activity is considered a major driver of aging, and the genetic or pharmacological suppression of the mTOR pathway has been demonstrated to prolong lifespan across multiple species [74]. mTOR complex 1 (mTORC1) and its downstream signaling pathways play a critical role in the aging process. The targeted inhibition of mTORC1 activity has the potential to treat aging-related diseases [75]. Yang et al. co-incubated various doses of MSCs–Exos with neonatal mouse follicles and observed an increase in phosphorylated mTOR expression in the MSC–Exo group. This activation of mTOR signaling promoted follicular development [76]. A further analysis revealed that miR-146a-5p and miR-21-5p in MSCs–Exos can activate the phosphatidylinositol 3-kinase (PI3K) and mTOR signaling pathway, thereby regulating the activation of neonatal mouse follicles [76]. In osteoarthritis, miR-100-5p carried by MSCs–Exos can target and inhibit mTOR luciferase activity, thereby suppressing mTOR signaling and protecting articular cartilage [77]. Qu et al. isolated primary MSCs from the umbilical cord and co-cultured their exosome-derived miR-126-3p with ovarian granulosa cells. The results showed that miR-126-3p enhances proliferative capacity and inhibits apoptosis by downregulating phosphoinositide-3-kinase regulatory subunit 2 (PIK3R2), thereby activating the PI3K/AKT/mTOR pathway [78]. MSC–Exo treatment significantly reduces ovarian damage in rats with premature ovarian failure, exerting anti-aging effects [78].

### 4.3. Sirtuins Signaling Pathway

Sirtuins are a class of NAD^+^-dependent deacetylases that play a critical role in gene regulation, apoptosis, energy metabolism, and aging-related diseases [79,80]. Multiple studies have found that NAD^+^ levels gradually decline with age, leading to reduced sirtuin activity [79]. Notably, sirtuin 7 (SIRT7) expression and activity are significantly decreased in tissues of aged mice or rats, such as in the colon and lung tissues of aged mice [81,82] and the liver tissues of aged rats [83]. MSCs–Exos exert anti-aging effects by activating sirtuin activity. In an aged mouse model, an injection of MSCs–Exos improved age-related cognitive impairment. Further research revealed that in the MSC–Exo-treated group, the upregulation of SIRT1 inhibited ferroptosis in aged mice [84]. Insulin resistance is commonly observed in the elderly population, and research has demonstrated that bone-marrow-derived MSCs–Exos can inhibit SIRT1 activity by targeting miR-29b-3p [85]. Su et al. achieved a marked improvement in insulin resistance in elderly mice by selectively reducing the expression of miR-29b-3p in MSCs–Exos [85]. Ovarian premature failure is clinically irreversible, and studies have shown that MSCs–Exos demonstrate promising therapeutic effects in the treatment of ovarian premature failure. miR-17-5p plays a crucial regulatory role by inhibiting SIRT7 activity, downregulating the expression of PARP1, γH2AX, and XRCC6, and reducing ROS levels in a mouse model of ovarian premature failure [86].

### 4.4. Other Pathways

The secretion of the SASP is a hallmark of cellular senescence, capable of inducing secondary senescence and disrupting homeostasis [46]. The SASP is a major driver of age-related inflammation [87]. The targeted regulation of the SASP has promise as a therapeutic strategy for treating aging-related diseases [88]. Studies have shown that MSCs–Exos participate in the regulation of the SASP through the delivery of miRNAs. miR-146a plays a crucial role in regulating cellular senescence. Xiao et al. found that extracellular vesicles derived from MSCs can reduce the SASP secretion of aging endothelial cells induced by oxidative stress. Specifically, miR-146a inhibits the phosphorylation of the steroid receptor coactivator (Src) and downstream targets such as vascular endothelial cadherin and Caveolin-1, thereby alleviating endothelial cell aging and stimulating angiogenesis [89]. Li et al. administered MSCs–Exos from human umbilical cords via intraperitoneal injections into aging mice and found that they could restore ovarian function, promoting follicle development and hormone production. Sequencing revealed that MSCs–Exos are rich in miR-21-5p, which targets and regulates phosphatase and tensin homolog (PTEN) protein to inhibit apoptosis and maintain normal ovarian function [65]. Recent research has shown that the bone-marrow-derived MSC–Exosome-mediated transport of miR-29b-3p effectively inhibits the expression of MMPs in skin tissues [90]. Increased MMP activity can promote endothelial inflammation, such as EC senescence/apoptosis/necrosis, thrombosis, and dysfunction [91]. In photodamaged mice, suppressing MMPs can effectively counteract the onset of aging [92]. Due to the anti-aging effects demonstrated by miRNAs carried by MSCs–Exos, the targeted delivery of miRNAs based on their unique regulatory mechanisms has great potential for delaying the aging process and treating age-related diseases.

## 5. Research Progress on MSC–Exo miRNAs in Age-Related Diseases

**Figure 3:**
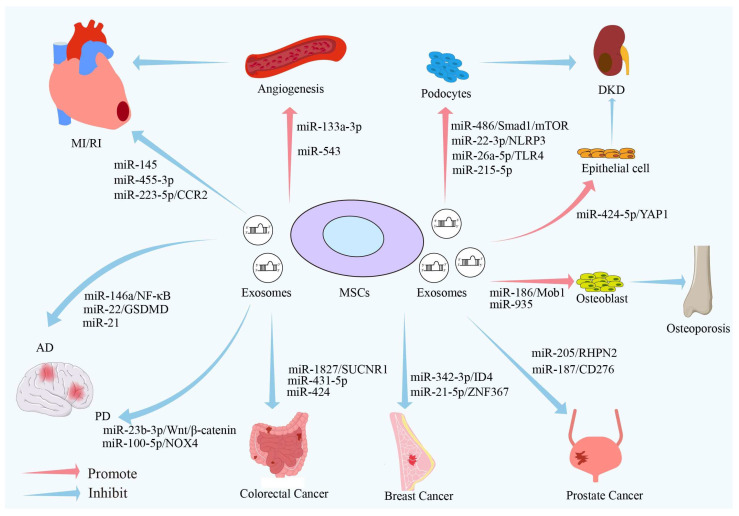
The role of MSC–Exo miRNAs in aging-related diseases. MSCs–Exos deliver miRNAs to target cells or organs, enhancing cellular activity and inhibiting disease progression. Figure 3 was drawn by the author of this article.

The aging process is driven by a range of internal and external factors and is closely associated with age-related conditions, degenerative diseases, the regulation of tumorigenesis, wound healing, and tissue repair mechanisms [93,94]. This includes cardiovascular diseases [95], diabetes [96], osteoporosis [97], cancer, and neurodegenerative diseases. As shown in Figure 3, the miRNAs carried by MSCs–Exos play a crucial role in the pathophysiology of the aforementioned age-related diseases.

### 5.1. MSC–Exo miRNAs and Cardiovascular Diseases

Cardiovascular diseases are a group of non-infectious disorders affecting the cardiovascular system, including arrhythmias, myocardial ischemia/reperfusion (MI/RI), atherosclerosis, and myocardial infarction, among others [98]. Due to factors such as unhealthy lifestyles and accelerated population aging, the incidence and mortality rates of cardiovascular diseases have been steadily increasing [99]. In 2019, approximately 17.9 million people worldwide died from cardiovascular diseases, accounting for 32% of global deaths [100]. Research evidence indicates that MSCs–Exos play a considerable regulatory role in the development of cardiovascular diseases. Analyses have found that Exos isolated from the culture medium of bone-marrow-derived MSCs are rich in miR-455-3p [101] and miR-145 [102], which can inhibit myocardial injury and cardiac cell dysfunction induced by MI/RI. Research indicates that miR-183-5p plays a key role in myocardial infarction. Zheng et al. discovered that MSCs–Exos have a protective effect against myocardial cell senescence in mice, with heme-treated MSCs–Exos demonstrating an enhanced therapeutic efficacy for myocardial infarction. MSCs–Exos can inhibit mitochondrial fission in cardiomyocytes and improve cellular conditions [103]. An analysis of miRNA expression differences between MSC-derived exosomes and those treated with heme revealed a significant upregulation of miR-183-5p in the heme-treated exosomes. miR-183-5p is capable of downregulating the expression of high mobility group box 1 (HMGB1), downregulating the extracellular signal-regulated kinase (ERK) pathway, and suppressing mitochondrial fission [103].

Arterial wall thickening and sclerosis caused by vascular aging, as well as abnormal changes in endothelial function, are major factors influencing the development of cardiovascular diseases [95]. Numerous studies have shown that MSCs–Exos participate in regulating angiogenesis by releasing miRNA, thereby alleviating cardiovascular diseases. For example, MSCs–Exos treated with macrophage migration inhibitory factor significantly promote cardiovascular regeneration in rats with myocardial infarction. Further miRNA sequencing analysis revealed a significant increase in the expression level of miR-133a-3p in the Exos. The overexpression of miR-133a-3p in vitro promotes the proliferation, migration, and angiogenesis of human umbilical vein endothelial cells, while decreasing cardiomyocyte apoptosis and fibrosis, leading to improved cardiac function in myocardial infarction rats [104]. Yang et al. found that MSCs–Exos can alleviate myocardial-infarction-induced damage, promote angiogenesis, and increase Ki-67 expression. However, this effect was removed when treated with an miR-543 inhibitor, indicating that MSCs–Exos exert their protective effects against myocardial infarction and promote angiogenesis through the delivery of miR-543 [105]. Tanshinone IIA (Tan IIA) is an effective drug for treating MI/RI and can reduce the occurrence of oxidative stress [106,107]. Li et al. developed a cell-free therapy for MI/RI using Tan IIA combined with MSCs–Exos. Their study found that exosomes derived from MSCs treated with Tan IIA significantly improved treatment outcomes for rats with MI/RI. The miRNA microarray results indicated that after Tan IIA treatment, miR-223-5p was significantly enriched in MSCs–Exos and targeted the inhibition of chemokine receptor 2 (CCR2) activity to reduce monocyte infiltration and enhance angiogenesis [108]. These studies suggest that MSCs–Exos can promote angiogenesis through their carried miRNAs, alleviate disease progression, and enhance therapeutic efficacy. Therefore, MSC–Exo-based therapies for cardiovascular diseases have considerable promise.

### 5.2. MSC–Exo miRNAs and DKD

The incidence and mortality rates of diabetes increase gradually after the age of 46, with the highest rates observed in individuals over 60. Additionally, the prevalence is higher in men than in women, making age and gender significant factors influencing diabetes [109]. It is estimated that the global number of individuals with diabetes will increase from 415 million in 2015 to 642 million by 2040 [110]. Diabetic kidney disease (DKD) is caused by microvascular damage induced by diabetes. Of the long-term complications associated with diabetes, DKD presents the heaviest burden in terms of economic costs and its effects on patients’ daily lives [111,112].

The primary clinical feature of DKD is increased urinary albumin excretion. The key factor leading to proteinuria is the detachment, apoptosis, or damage of podocytes [113,114]. Research has confirmed that miRNAs delivered by MSCs–Exos can be involved in the regulation of podocytes [115,116,117]. Jin et al. discovered that treating spontaneous diabetic mice with adipose-derived MSCs–Exos reduced urine protein, serum creatinine, and blood urea nitrogen levels. It was also found that MSCs–Exos could reduce podocyte damage and deaths induced by high glucose concentrations. An analysis revealed that miR-486 targets and inhibits the expression of Smad1, which further suppresses the activation of mammalian target of rapamycin (mTOR), leading to increased autophagy and reduced podocyte apoptosis [117]. Additionally, adipose-derived MSCs–Exos protect podocytes from damage in high-glucose environments by delivering miR-26a-5p, which targets Toll-like receptor 4 (TLR4), inhibits nuclear factor-κB (NF-κB) signaling, and reduces the expression of vascular endothelial growth factor (VEGF) [118]. Wang et al. reported that umbilical cord-derived MSCs–Exos have anti-inflammatory effects that protect podocytes in a high-glucose environment and inhibit their apoptosis. They further found that miR-22-3p in MSCs–Exos can suppress the expression of its target protein, NOD-like receptor pyrin domain-containing protein 3 (NLRP3) [116]. Additionally, renal tubular epithelial cells are integral to the pathophysiology of DKD. Chen et al. found that MSCs–Exos can inhibit the epithelial-to-mesenchymal transition and apoptosis of renal tubular epithelial cells. In vivo experiments demonstrated that MSCs–Exos can improve kidney function, while in vitro studies showed that the miR-424-5p enriched in Exos can target and protect renal tubular epithelial cells by suppressing the expression of Yes-associated protein 1 (YAP1) [119].

### 5.3. MSC–Exo miRNAs and Osteoporosis

Osteoporosis is a bone metabolic disorder, and over 200 million people worldwide are affected by it. Around half of women and one-fifth of men will suffer from osteoporotic fractures after turning 50 [120]. The prevalence of osteoporosis is also associated with factors such as gender, age, smoking status, and the presence of previous fractures [121]. Osteoporosis arises from an imbalance between osteoclast-mediated bone resorption and osteoblast-driven bone formation, resulting in bone loss and structural degradation [120,122]. Co-culturing osteoblasts with exosomes from different sources revealed that MSCs–Exos promote osteoblast proliferation more effectively than exosomes from osteoblasts themselves. Among these, miR-2110 and miR-328-3p are important osteogenic regulatory miRNAs [123]. Zhang et al. reported that bone-marrow-derived MSCs–Exos deliver miR-935 to osteoblasts, inhibiting signaling pathways and reducing the level of signal transducer and activator of transcription 1 (STAT1). The downregulation of STAT1 promotes osteoblast proliferation and differentiation and increases alkaline phosphatase activity in mineralized nodules [124]. Li et al. also found that miR-186 in bone marrow MSCs–Exos plays a central role in bone formation. Mechanistic studies revealed that miR-186 binds to and inhibits the expression of kinase suppressor of Ras 1 (Mob1), regulating the Hippo signaling pathway and promoting bone formation in ovariectomized rats [125].

### 5.4. MSC–Exo miRNAs and Cancer

Aging is one of the most significant risk factors for various types of cancer, with the incidence of cancer increasing with age [126]. According to data from the National Cancer Center, there were approximately 4.064 million new cases of malignant tumors in 2016, primarily including lung cancer, colorectal cancer, stomach cancer, liver cancer, and breast cancer [127]. MSCs–Exos play a crucial role in tumor growth, angiogenesis, the tumor microenvironment, and drug resistance [128]. Exploring their specific mechanisms in cancer progression is essential for targeted cancer therapies.

Prostate cancer is a prevalent and significant malignancy in males and ranks among the top causes of cancer-related mortality globally [129,130]. Research has shown that co-culturing prostate cancer cells with Exos containing miR-205 mimics derived from MSCs effectively inhibits cancer cell invasion, migration, and proliferation. An analysis revealed that miR-205 inhibits the malignant progression of prostate cancer by suppressing its target gene rhophilin rho GTPase binding protein 2 (RHPN2). Li et al. found that MSCs–Exos carrying miR-187 help suppress the malignant characteristics, tumorigenic ability, and metastasis of prostate cancer. The upregulation of miR-187 inhibits CD276 expression and the Janus kinase 3 (JAK3)-STAT3-Slug signaling pathway [130].

Breast cancer is among the most common cancers worldwide, representing 30% of cancer diagnoses in women. Yu et al. reported that miR-342-3p is highly expressed in MSCs–Exos and is associated with breast cancer metastasis and chemoresistance. miR-342-3p exerts its potential anti-cancer effect by inhibiting the expression of DNA-binding inhibitor 4 (ID4), which downregulates the resistance of breast cancer cells to doxorubicin, fluorouracil, and cisplatin [131].

MSCs–Exos also play a role in regulating the growth and metastatic ability of colorectal cancer. Chen et al. observed that the co-incubation of MSCs–Exos with colorectal cancer cells led to a decrease in cell proliferation, migration, and invasion. They also found that transfection with an miR-1827 mimic downregulated succinate receptor 1 (SUCNR1) expression and inhibited the M2 polarization of macrophages, thereby preventing liver metastasis in patients with colorectal cancer [132]. Additionally, other studies have found that the overexpression of miR-431-5p in human umbilical cord MSCs–Exos also inhibits the aggressive progression of colorectal cancer and may act as a potential biomarker for the condition [133]. These studies reveal the significant role of MSCs–Exos and their carried miRNAs in cancer proliferation, metastasis, and drug resistance, further suggesting that MSCs–Exos have promise as a therapeutic strategy for cancer.

### 5.5. MSC–Exo miRNAs and Neurodegenerative Diseases

Neurodegenerative diseases related to aging include Parkinson’s disease (PD) and Alzheimer’s disease (AD). PD is caused by a reduction in dopamine in the substantia nigra, loss of neurons, and accumulation of Lewy bodies in other brain regions [134]. Geng et al. found that the level of miR-23b-3p is elevated in MSCs–Exos, and the upregulation of miR-23b-3p can activate the Wnt/β-catenin signaling pathway, promoting autophagy in PD cell models. Additionally, in a PD rat model, MSCs–Exos were shown to regulate the Wnt signaling pathway through miR-23b-3p, thereby enhancing neuronal autophagy. Furthermore, MSCs–Exos can also modulate oxidative stress in PD cells, maintaining the function of the nigrostriatal system [135]. He et al. injected MSCs–Exos into a PD mouse model via the tail vein and observed that this treatment effectively improved motor function in the PD mice, slowed the loss of dopaminergic neurons, and reduced oxidative stress damage associated with PD. After a deeper analysis, it was found that miR-100-5p, which is enriched in MSCs–Exos, exerts a protective effect on PD cells by directly targeting the 3′ UTR of NADPH oxidase 4 (NOX4) [136]. These studies provide a strong experimental foundation for MSCs–Exos as a therapeutic strategy for PD.

AD is the most common cause of dementia in the elderly, accounting for 60% to 80% of all dementia cases, and its incidence continues to rise [137]. Currently, the main treatments for AD include cholinesterase inhibitors and N-methyl-D-aspartate (NMDA) receptor antagonists. However, these medications are aimed at prolonging life and improving patients’ quality of life, and they cannot prevent the onset or progression of the disease [138]. Research indicates that MSCs–Exos can effectively alleviate symptoms of AD and enhance therapeutic efficacy. MSCs from bone marrow transfer miR-146a to hippocampal astrocytes through secreted exosomes, reducing the expression of NF-κB and exerting anti-inflammatory effects [139]. Cui et al. reported that hypoxia-treated MSCs–Exos can improve cognitive and memory functions in AD mice. MSCs–Exos also promote the expression of anti-inflammatory molecules such as interleukin-4 (IL-4) and IL-10. A co-culture with these exosomes significantly upregulated the expression of miR-21 in the brains of AD mice, which reduced plaque deposition and decreased levels of tumor necrosis factor alpha (TNF-α) and IL-1β [140]. Chen et al. used MSCs–Exos to transfer miR-22 to AD mice and found that this approach decreased the expression of inflammatory factors and increased the survival rate of neural cells. miR-22 can suppress pyroptosis by targeting gasdermin D (GSDMD) and improve memory and motor skills in AD mice by inhibiting inflammatory responses [141].

## 6. MSCs–Exos for Targeted miRNA Delivery in Treating Aging-Related Diseases

The use of exosomes as nanocarriers for drug delivery has been extensively studied. Exosomes can precisely deliver therapeutic agents to disease sites, enhancing treatment efficacy while minimizing side effects [142]. miRNAs exert biological activity by participating in multiple signaling pathways, including apoptosis, inflammation, autophagy, and oxidative stress. Exosomes carrying miRNAs can effectively suppress disease progression [143,144]. In a humanized acute myeloid leukemia (AML) mouse model, exosomes loaded with miR-34c-5p effectively eliminated leukemia stem cells and inhibited the progression of AML [143]. Cao et al. prepared MSCs–Exos enriched with therapeutic miRNAs and injected them into rats with osteoarthritis. The study found that MSCs–Exos inhibited the p53 signaling pathway, reduced MMP-13 fluorescence activity, and improved the cartilage lesions of the rats [71]. Zhao et al. synthesized engineered exosomes enriched with miR-199a-3p through MSCs, which were shown to induce cartilage regeneration [145]. Numerous animal studies have demonstrated that MSCs–Exos can serve as carriers for the targeted delivery of miRNAs in the treatment of aging-related diseases (Table 1). These studies suggest that targeted delivery of therapeutic miRNAs via MSCs–Exos can effectively inhibit disease progression, representing a promising therapeutic approach.

Although these studies demonstrate that the delivery of miRNAs via MSCs–Exos significantly alleviates the disease burden and inhibits disease progression in animal models, the mechanisms of many miRNAs remain undetermined. Studies using high-throughput sequencing combined with low-throughput experimental validation have identified 2300 mature miRNAs in human cell lines [160]. With the advancement and refinement of sequencing standards and related technologies, the number of identified miRNAs continues to grow. Currently, research on miRNAs in aging-related diseases is limited. Extensive experimental and clinical studies are needed to uncover new miRNA regulatory functions and mechanisms and to determine which miRNAs have therapeutic potential and which promote disease onset and progression.

## 7. The Pro-Aging Potential of Exosomal miRNAs

The regulatory functions of miRNAs not only inhibit cellular senescence but also promote aging processes. Yin et al. demonstrated that macrophage-derived exosomes drive tubular cell senescence by delivering miR-155 [161]. Senescence-associated exosomes promote the onset of aging by transferring various regulatory factors through autocrine or paracrine mechanisms [162]. Li et al. discovered that exosomes secreted by senescent endothelial cells promote the senescence of skin fibroblasts by targeting miR-767 to suppress TGF-beta activated kinase 1 binding protein 1 (TAB1) expression, thereby inhibiting fibroblast proliferation and increasing apoptosis [163]. In exosomes derived from bone marrow MSCs of aged rats, the expression of miR-31a-5p was significantly upregulated. Further studies revealed that miR-31a-5p promotes the expression of senescence-associated markers by inhibiting E2F transcription factor 2 (E2F2) activity, which leads to increased osteoclast activity and ultimately results in osteoporosis [164]. Additionally, Su et al. found that exosomes released by bone marrow MSCs from aged mice regulate the function of adipocytes, myocytes, and hepatocytes through miR-29b-3p, leading to the development of age-related insulin resistance in these mice [85]. These studies suggest that MSC–Exos may also exert pro-aging effects through the action of miRNAs.

## 8. Challenges, Conclusions, and Future Prospects

Aging is a complex physiological process that can be induced by intrinsic or extrinsic factors, including reactive ROS, ultraviolet radiation, environmental mutagens, and chemical agents [2]. MSCs, as multipotent stem cells, possess potent immunoregulatory abilities that can effectively mitigate the progression of aging and aging-related diseases [165]. Therefore, studying the role of MSCs and their derivatives in aging provides new insights for the development of therapeutic strategies for aging-related diseases.

Exosomes, as extracellular vesicles serving as intercellular communication tools, can selectively deliver cargo. Multiple studies have found that MSCs–Exos effectively delay aging, with their carried miRNAs involved in regulating cellular aging processes. In clinical trials, exosomes not only serve as crucial carriers for biomarkers but also function as a drug delivery system with enhanced targeting capabilities and lower cytotoxicity [166]. miRNAs can serve as small-molecule drugs for the targeted treatment of diseases, such as miRNA mimics, inhibitors, and others [167]. Therefore, therapeutic strategies targeting miRNAs in exosomes have promise as new avenues for treating age-related diseases. However, the large-scale production and extraction of exosomes face significant challenges. Firstly, unlike chemical drugs, exosomes contain complex components, making industrial-scale production difficult. Secondly, the lack of efficient and standardized methods for exosome extraction results in low separation purity. Streamlining exosome extraction and detection times and improving exosome purity will aid in advancing research and development related to aging and age-related diseases. MSCs from different sources exhibit heterogeneity at both the molecular and functional levels [168], with their secreted exosomes displaying variability in their enriched components and the types of microRNAs they carry. Furthermore, research on MSCs–Exos remains limited. Exosomes from MSCs with different origins exhibit heterogeneity in their enriched components and the types of miRNAs they carry. Which miRNAs are enriched in MSCs–Exos? How do these miRNAs participate in regulating cellular aging and what roles do they play? These questions require further investigation.

In conclusion, MSCs–Exos regulate aging-related genes and signaling pathways through miRNA involvement, exerting effects against aging and age-related diseases. Aging-related disease animal models have shown that the targeted delivery of miRNA via MSCs–Exos can effectively improve aging-related diseases and exert anti-aging effects. Studying the specific roles of MSCs–Exos and the miRNAs they carry in cellular aging and related diseases helps unravel the mysteries of aging, offering new insights for delaying aging and treating age-related conditions.

## Figures and Tables

**Table 1:** MSCs–Exos as miRNA delivery system in aging-related diseases.

miRNAs	Diseases	Exosomal Expression	Target	Function	Ref.
miR-199a-3p	OA	↑	Downregulate mTOR	Diseases suppressor	[145]
miR-100-5p	OA	↑	Downregulate mTOR	Diseases suppressor	[77]
miR-302	MI/RI	↑	Downregulate YAP	Diseases suppressor	[146]
miR-155	Diabetic	↓	Downregulate FGF7	Diseases suppressor	[147]
miR-155-5p	OA	↑	Downregulate RUNX2	Diseases suppressor	[148]
miR-126-3p	Diabetic	↑	Promote p-AKT and p-ERK1/2	Diseases suppressor	[149]
miR-143-3p	MI/RI	↑	Downregulate CHK2	Diseases suppressor	[150]
miR-146a-5p	Diabetic	↑	Downregulate TRAF6	Diseases suppressor	[151]
miR-22	AD	↑	Downregulate GSDMD	Diseases suppressor	[141]
miR-19b-3p	Osteoporosis	↑	Downregulate PTEN	Diseases suppressor	[152]
miR-100a-5p	PD	↑	Downregulate NOX4	Diseases suppressor	[136]
miR-935	Osteoporosis	↑	Downregulate STAT1	Diseases suppressor	[124]
miR-205	Prostate cancer	↑	Downregulate RHPN2	Diseases suppressor	[153]
miR200b-3p	Diabetic	↑	Downregulate SYDE1	Diseases suppressor	[154]
miR-199a	HCC	↑	Downregulate mTOR	Diseases suppressor	[155]
miR-133b	Glioma	↑	Downregulate EZH2	Diseases suppressor	[156]
miR-144	NSCLC	↑	Downregulate CCNE1 and CCNE2	Diseases suppressor	[157]
miR-375	ESCC	↑	Downregulate ENAH	Diseases suppressor	[158]
miR-16-5p	DN	↑	Downregulate VEGFA	Diseases suppressor	[159]
miR-125b-5p	MI	↑	Downregulate p53 and BAK1	Diseases suppressor	[72]

HCC: Hepatocellular carcinoma; NSCLC: Non-small cell lung cancer; ESCC: Esophageal squamous cell carcinoma; YAP: Yes1 associated transcriptional regulator; FGF7: fibroblast growth factor 7; RUNX2: RUNX family transcription factor 2; ERK1/2: Extracellular signal-regulated kinase 1/2; CHK2: checkpoint kinase 2; TRAF6: TNF receptor-associated factor 6; GSDMD: gasdermin D; NOX4: NADPH oxidase 4; STAT1: signal transducer and activator of transcription 1; RHPN2: rhophilin Rho GTPase binding protein 2; SYDE1: synapse defective Rho GTPase homolog 1; EZH2: enhancer of zeste 2 polycomb repressive complex 2 subunit; CCNE1: cyclin E1; CCNE2: cyclin E2; ENAH: ENAH actin regulator; VEGFA: vascular endothelial growth factor A; BAK1: BCL2 antagonist/killer 1; ↑: indicates upregulation; ↓: indicates downregulation.

## Abbreviations

MSCs: mesenchymal stem cells; Exos: exosomes; ILVs: intraluminal vesicles; MVBs: multivesicular bodies; SASP: senescence-associated secretory phenotype; ROS: reactive oxygen species; MMPs: matrix metalloproteinases; MTOR: Mechanistic target of rapamycin kinase; HCC: Hepatocellular carcinoma; NSCLC: Non-small cell lung cancer; ESCC: Esophageal squamous cell carcinoma; YAP: Yes1 associated transcriptional regulator; FGF7: fibroblast growth factor 7; RUNX2: RUNX family transcription factor 2; ERK1/2: Extracellular signal-regulated kinase 1/2; CHK2: checkpoint kinase 2; TRAF6: TNF receptor associated factor 6; GSDMD: gasdermin D; NOX4: NADPH oxidase 4; STAT1: signal transducer and activator of transcription 1; RHPN2: rhophilin Rho GTPase binding protein 2; SYDE1: synapse defective Rho GTPase homolog 1; EZH2: enhancer of zeste 2 polycomb repressive complex 2 subunit; CCNE1: cyclin E1; CCNE2: cyclin E2; ENAH: ENAH actin regulator; VEGFA: vascular endothelial growth factor A; BAK1: BCL2 antagonist/killer 1.
